# zipHMMlib: a highly optimised HMM library exploiting repetitions in the input to speed up the forward algorithm

**DOI:** 10.1186/1471-2105-14-339

**Published:** 2013-11-22

**Authors:** Andreas Sand, Martin Kristiansen, Christian NS Pedersen, Thomas Mailund

**Affiliations:** 1 Bioinformatics Research Centre, Aarhus University, Aarhus, Denmark; 2 Department of Computer Science, Aarhus University, Aarhus, Denmark

## Abstract

**Background:**

Hidden Markov models are widely used for genome analysis as they combine ease of modelling with efficient analysis algorithms. Calculating the likelihood of a model using the forward algorithm has worst case time complexity linear in the length of the sequence and quadratic in the number of states in the model. For genome analysis, however, the length runs to millions or billions of observations, and when maximising the likelihood hundreds of evaluations are often needed. A time efficient forward algorithm is therefore a key ingredient in an efficient hidden Markov model library.

**Results:**

We have built a software library for efficiently computing the likelihood of a hidden Markov model. The library exploits commonly occurring substrings in the input to reuse computations in the forward algorithm. In a pre-processing step our library identifies common substrings and builds a structure over the computations in the forward algorithm which can be reused. This analysis can be saved between uses of the library and is independent of concrete hidden Markov models so one preprocessing can be used to run a number of different models.

Using this library, we achieve up to 78 times shorter wall-clock time for realistic whole-genome analyses with a real and reasonably complex hidden Markov model. In one particular case the analysis was performed in less than 8 minutes compared to 9.6 hours for the previously fastest library.

**Conclusions:**

We have implemented the preprocessing procedure and forward algorithm as a C++ library, zipHMM, with Python bindings for use in scripts. The library is available at http://birc.au.dk/software/ziphmm/.

## Background

Hidden Markov models (HMMs) are a class of statistical models for sequential data with an underlying hidden structure. They were first introduced to bioinformatics in the late 1980s [1] and have since then been used in a wide variety of applications, for example for gene finding [2], modelling of protein structures [3,4], sequence alignment [5] and phylogenetic analysis [6-9]. Because of their computational efficiency HMMs are one of the few widely used statistical methodologies that are feasible for genome wide analysis where sequences often are several hundred million characters long. With data sets of this size, however, analysis time is still often measured in hours and days. Improving on the performance of HMM analysis is therefore important to keep up with the quickly growing amount of biological sequence data to be analysed, and to make more complex analyses feasible.

The most time consuming part of using hidden Markov models is often parameter fitting, since the likelihood of a model needs to be computed repeatedly when optimising the parameters. Depending on the optimisation strategy, this means that the forward algorithm (or both the forward and the backward algorithm) will be evaluated in potentially hundreds of points in parameter space. Optimising the forward algorithm is therefore the most effective strategy for efficient HMM implementations.

The forward algorithm can be seen as a sequence of vector-matrix operations along an input sequence. This, however, can be rewritten as a sequence of matrix-matrix operations. This replaces an $O(n^{2})$ time vector-matrix operation with an $O(n^{3})$ time matrix-matrix operation but opens up possibilities for changing the evaluation order since different parts of the computation now can be handled independently. This then makes it possible to reuse computations whenever the input contains repeated substrings [10] or to parallelise the algorithm across a number of independent threads [11].

The main contribution of this paper is a software library that uses both of these ideas to greatly speed up the forward algorithm. We present a preprocessing of the observed sequence that finds common substrings and constructs a data structure that makes the evaluation of the likelihood close to two orders of magnitude faster (not including the preprocessing time). The preprocessing of a specific sequence can be saved and later reused in the analysis of a different HMM topology. The algorithms have been implemented in a C++ library, zipHMMlib, available at http://birc.au.dk/software/ziphmm/. The library also provides an interface to the Python programming language.

Much of the theory used in zipHMMlib was also developed by Lifshits et al. [10], but while they developed the theory in the context of the Viterbi algorithm, where the preprocessing cannot be reused, we concentrate on the forward algorithm and introduce a data structure to save the preprocessing for later reuse. We furthermore extend the theory to make the computations numerically stable and introduce practical measures to make the algorithm run fast in practice and make the library accessible.

Our implementation is tested on simulated data and on alignments of chromosomes from humans with chimpanzees, gorillas and orangutans analysed with the CoalHMM framework [7,8,12], a framework which uses changes in coalescence trees along a sequence alignment to make inference in population genetics and phylogenetics and which has been used in a number of whole-genome analyses [13-16]. Using an “isolation-with-migration” CoalHMM [17], we train the model using the Nelder-Mead-simplex algorithm and measure the preprocessing time and total optimisation time. Looking at the time required to perform the entire training procedure, we achieve up to 78 times shorter wall-clock time compared to the previously fastest implementation of the forward algorithm. Even for data of high complexity and with few repetitions we achive a speedup of a factor 4.4.

## Implementation

### Hidden Markov models

A Hidden Markov Model (HMM) describes a joint probability distribution over an observed sequence $Y_{1:T}=y_{1}y_{2}\ldots y_{T}\in O^{*}$ and a hidden sequence $X_{1:T}=x_{1}x_{2}\ldots x_{T}\in {ℋ}^{*}$, where  and  are finite alphabets of observables and hidden states, respectively. The hidden sequence is a realisation of a Markov process which explains hidden properties of the observed data. We can formally define an HMM [18] as consisting of: 

•$ℋ=\{h_{1},h_{2},\ldots ,h_{N}\}$, a finite alphabet of hidden states;

•$O=\{o_{1},o_{2},\ldots ,o_{M}\}$, a finite alphabet of observables;

•a vector *Π*=(*π*_*i*_)_1≤*i*≤*N*_, where *π*_*i*_= Pr(*x*_1_=*h*_*i*_) is the probability of the model starting in hidden state *h*_*i*_;

•a matrix *A*={*a*_*ij*_}_1≤*i*, *j*≤*N*_, where *a*_*ij*_= Pr(*x*_*t*_=*h*_*j*_|*x*_*t*−1_=*h*_*i*_) is the probability of a transition from state *h*_*i*_ to state *h*_*j*_;

•a matrix $B={\left\{b_{\text{ij}}\right\}}_{1\leq i\leq N}^{1\leq j\leq M}$, where *b*_*ij*_= Pr(*y*_*t*_=*o*_*j*_|*x*_*t*_=*h*_*i*_) is the probability of state *h*_*i*_ emitting *o*_*j*_.

An HMM is parameterised by *π*, *A* and *B*, which we will denote by *λ*=(*π*,*A*,*B*).

### The classical forward algorithm

The forward algorithm [18] finds the probability of observing a sequence *Y*_1:*T*_ in a model *λ* by summing the joint probability of the observed and hidden sequences for all possible hidden sequences: $\mathrm{Pr}\left(Y_{1:T}\;|\;\lambda \right)=\underset{x_{1:T}}{\sum }$ Pr(*Y*_1:*T*_,*X*_1:*T*_=*x*_1:*T*_ | *λ*). This sum is normally computed by first filling out a table, *α*, with entries *α*_*t*_(*x*_*t*_)= Pr(*Y*_1:*t*_,$\left(X_{t}=x_{t}\;|\;\lambda \right)=\underset{x_{1:t-1}}{\sum }\mathrm{Pr}\left(Y_{1:t},X_{1:t}=x_{1:t}\;|\;\lambda \right)$ column by column from left to right, using the recursion

(1) $$\begin{array}{cc} \alpha_{1}(x_{1}) & =\pi_{x_{1}}b_{x_{1},y_{1}}\\ \alpha_{t}(x_{t}) & =b_{x_{t},y_{t}}\;\underset{x_{t-1}}{\sum }\;\alpha_{t-1}(x_{t-1})\;a_{x_{t-1},\;x_{t}}. \end{array}$$

After filling out *α*, Pr(*Y*_1:*T*_ | *λ*) can be computed as $\mathrm{Pr}\left(Y_{1:T}\;|\;\lambda \right)=\underset{x_{T}}{\sum }\alpha_{T}(x_{T})$.

### The algorithm as linear algebra

In the classical forward algorithm, we compute the columns of *α* from left to right by the recursion in equation (1). If we can compute the last one of these columns, *α*_*T*_, efficiently, we can compute $\mathrm{Pr}\left(Y_{1:T}\;|\;\lambda \right)=\underset{x_{T}}{\sum }\alpha_{T}(x_{T})$. Now let *α*_*t*_ be the column vector containing the *α*_*t*_(*x*_*t*_)’s:

$$\alpha_{t}=\left[\begin{array}{c} \alpha_{t}(h_{1})\\ \alpha_{t}(h_{2})\\ \mathrm{...}\\ \alpha_{t}(h_{N}) \end{array}\right],$$

let $B_{o_{i}}$ be the diagonal matrix, having the emission probabilities of *o*_*i*_ on the diagonal:

$$B_{o_{i}}=\left[\begin{array}{c} b_{1,o_{i}}\\ \;b_{2,o_{i}}\\ \;\cdots \\ \;b_{N,o_{i}} \end{array},\;\right]\;,$$

and let

(2) $$C_{o_{i}}=B_{o_{i}}A^{*}\;\text{and}\;C_{1}=B_{y_{1}}\pi ,$$

where *A*^∗^ is the transpose of *A*. Then *α*_*t*_ can be computed using only $C_{y_{t}}$ and the previous column vector *α*_*t*−1_:

(3) $$\alpha_{t}=C_{y_{t}}\alpha_{t-1}=C_{y_{t}}C_{y_{t-1}}\cdots C_{y_{2}}C_{1}.$$

**Figure 1:**
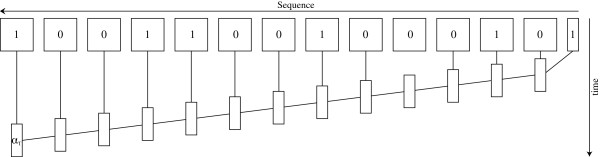
**Classical approach to the forward algorithm.** The classical forward algorithm, as described by Rabiner [18]. The rectangles represent matrices and vectors. The black lines denote matrix-vector multiplications. The top row is the $C_{o_{i}}$ matrices. *α*_*i*_ is obtained from *α*_*i*−1_ and $C_{o_{i}}$. Note that the input sequence is inverted to illustrate that the series of matrix-vector multiplication should be carried out from right to left.

Thus the classical forward algorithm can be described as a series of matrix-vector multiplications of length *T*−1 as illustrated in Figure 1. The classical forward algorithm corresponds to computing this from right to left, but since matrix-matrix multiplication is associative the product can be computed in any order. Since repeated substrings corresponds to repeated matrix-matrix multiplications, running time can be improved by reusing shared expressions [10,11]. In the following sections we will show how we precompute a grouping of the terms based on *Y*_1:*T*_ in order to minimise the workload in the actual computation of the likelihood.

### Exploiting repetitions in the observed sequence

Let $o_{i}o_{j}\in O\times O$ be the pair of symbols occurring most often without overlap in *Y*_1:*T*_, and let $n_{o_{i}o_{j}}$ be the number of occurrences. We can then reduce the length of *Y*_1:*T*_ with $n_{o_{i}o_{j}}$ characters by introducing a new symbol *o*_*M*+1_ and replacing all occurrences of *o*_*i*_*o*_*j*_ by this symbol. To mimic this in the computation described above, we introduce a new *C* matrix:

$$C_{o_{M+1}}=C_{o_{i}}C_{o_{j}}.$$

Now notice that we only need to compute this matrix once and substitute it for all occurrences of $C_{o_{i}}C_{o_{j}}$ in equation (3). Hence we can save $n_{o_{i}o_{j}}$ matrix-vector multiplications by introducing one matrix-matrix multiplication, potentially saving us a large amount of work.

These observations suggest that we can split the computation of the likelihood of a given observed sequence in a preprocessing of the sequence and in the actual computation of the likelihood. In the preprocessing phase we compress the observed sequence by repeatedly finding the most frequent pair of symbols *o*_*i*_*o*_*j*_ in the current sequence and replacing all occurrences of this pair by a new symbol. This is repeated until $n_{o_{i}o_{j}}$ becomes too small to gain a speedup (see next section). The result is a sequence $Y_{1:T^{\prime }}^{\prime }$ over a new alphabet $O^{\prime }=\{o_{1},o_{2},\ldots ,o_{M},o_{M+1}=(l_{1},r_{1}),o_{M+2}=(l_{2},r_{2}),\ldots ,o_{M^{\prime }}=(l_{M^{\prime }-M},r_{M^{\prime }-M})\}$, where *l*_*i*_,*r*_*i*_∈{*o*_1_,*o*_2_,…*o*_*i*−1_}. This compression will be identical independent of the HMM, meaning that we can save it along with the observed sequence and reuse it for any HMM.

The actual computation of the likelihood is then split in two stages. In the first stage we compute *C*_1_ and $C_{o_{i}}$ for *i*=1,…,*M* using (2). We then compute $C_{o_{i}}$ for increasing *i*=*M*+1,…,*M*^′^ by $C_{o_{i}}=C_{l_{i}}C_{r_{i}}$. In the second stage, we compute *α*_*T*_ by

$$\alpha_{T}=C_{y_{T^{\prime }}^{\prime }}C_{y_{T^{\prime }-1}^{\prime }}\cdots C_{y_{2}^{\prime }}C_{1}.$$

**Figure 2:**
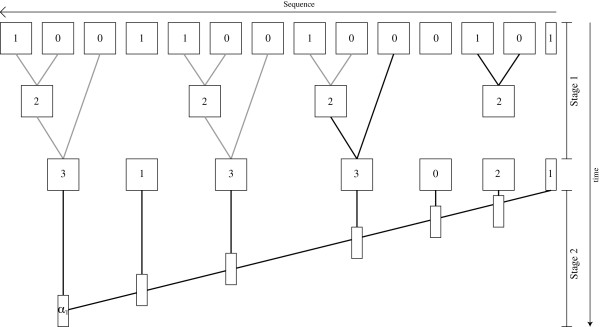
**Reusing common expressions to speed up the forward algorithm.** The actual computation of the likelihood of the observed sequence 10100010011001 given a specific HMM. The rectangles represent matrices and vectors and lines between them represent dependencies. In stage 1 the $C_{o_{i}}$ matrices are computed. The solid black lines show the amount of work performed, while the grey lines show the amount of work saved due to redundancy. *C*_2_ is for example computed as the product *C*_1_*C*_0_, and this multiplication is saved three times. In the second stage *α*_*T*_ is computed from the compressed sequence and the $C_{o_{i}}$ matrices. As in Figure 1*α*_*i*_ is computed from *α*_*i*−1_ and $C_{o_{i}}$.

This is illustrated in Figure 2 where the actual computation is drawn in solid black, while the saved work due to redundancy is shown in gray.

### Compression stopping criterion

**Figure 3:**
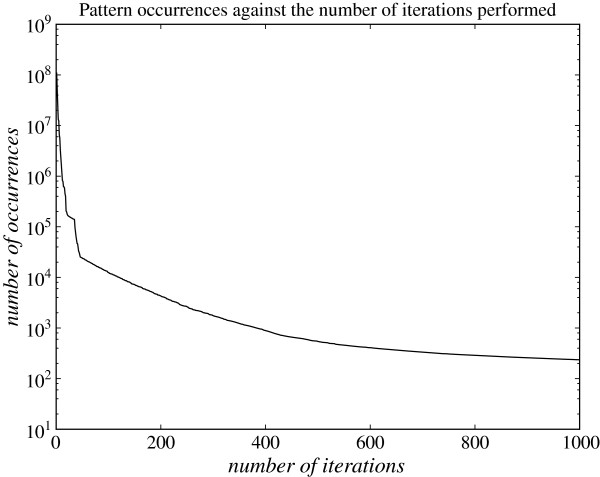
**Number of pattern occurrences against the number of iterations performed.** The number of occurrences of the most frequent pair of symbols plotted on a log-scale against the number of iterations performed of the preprocessing procedure on an alignment of the human and chimpanzee chromosome 1 encoded using the alphabet {0,1,2} for identical sites, differing sites or missing data, respectively.

While the first iterations of the preprocessing procedure compress the sequence very effectively, the last iterations do not decrease the sequence length by much, since most pairs are uncommon when more characters are introduced. This is illustrated in Figure 3, where we see that the number of occurrences of the most frequent pair of symbols decreases superexponentially as a function of the number of iterations performed on an alignment of the human and chimpanzee chromosome 1. This means that we potentially save a lot of time on the likelihood computation by performing the first iterations, but as the slope of the curve increases towards 0 we risk to spend a long time on the preprocessing and save very little time on the actual likelihood computation. To overcome this problem, we do not compress the input sequence all the way down to a single character. Assume we know that the preprocessing will not be reused for an HMM with less than *N*_*min*_ states, and let *t*_*mv*_ be the time required for an (*N*_*min*_×*N*_*min*_)×*N*_*min*_ matrix-vector multiplication and *t*_*mm*_ be the time required for an (*N*_*min*_×*N*_*min*_)×(*N*_*min*_×*N*_*min*_) matrix-matrix multiplication. In iteration *i* of the preprocessing we replace the most frequent pair of two symbols in the current sequence and find the most frequent pair of two symbols in the resulting sequence.

Thus if *p*_*i*_ is the number of occurrences of the pair found in iteration *i*, *pre*_*i*_ is the time required for iteration *i*, and *e* is an estimate (given by the user) of the number of times the preprocessing is going to be reused (for example in a number of training procedures each calling forward several times), then, assuming that the matrix-vector multiplications and matrix-matrix multiplications dominate the runtime of the actual likelihood computations, the amount of time that is saved by running iteration *i* is *e*(*t*_*mv*_*p*_*i*−1_−*t*_*mm*_)−*pre*_*i*_, as we save *p*_*i*−1_ matrix-vector multiplications in each likelihood computation, and we do this by introducing one new matrix-matrix multiplication. This means that the optimal time to stop the preprocessing is before iteration *j*, where *j* is the minimal value of *i* making *e*(*t*_*mv*_*p*_*i*−1_−*t*_*mm*_)−*pre*_*i*_ less than or equal to 0. However, we do not know *pre*_*i*_ before iteration *i* has been completed, but we can estimate it by *pre*_*i*−1_. Thus we stop the preprocessing just before iteration *j*, where *j* is the minimal value of *i* making *e*(*t*_*mv*_*p*_*i*−1_−*t*_*mm*_)−*pre*_*i*−1_ less than or equal to 0.

The values *t*_*mv*_ and *t*_*mm*_ are measured prior to the preprocessing, whereas the user has to supply an estimate, *e*, of the number of reuses of the preprocessing and *N*_*min*_. If a single value of *N*_*min*_ can not be determined, we allow the user to specify a list of state space sizes $\left(N_{\min}^{1},N_{\min}^{2},\mathrm{...}\right)$ for which he wants the preprocessing to be saved. If no *N*_*min*_ values are provided, the compression is stopped whenever *p*_*i*_=*p*_*i*−1_ for the first time.

### Numerical stability

All our matrices contain probabilities, so all entries are between 0 and 1. This means that their products will tend towards 0 exponentially fast. The values of these products will normally be stored in one of the IEEE 754 floating-point formats. These formats have limited precision, and if the above was implemented naïvely the results would quickly underflow.

If we can make do with log(Pr(*Y*_1:*T*_ | *λ*)), we can prevent this underflow by repeatedly rescaling the matrices, much in the same way as the columns are rescaled in the numerically stable version of the classical forward algorithm [18]. To make this work in our case, we will normalise the results of each matrix-matrix multiplication or matrix-vector multiplication we do throughout the algorithm and work with the normalised matrices instead. We first take care of the rounding errors that can propagate through the first stage of the likelihood computations (depicted in the top part of Figure 3) if the dependency graph between the new symbols is deep. Let

$$c_{o_{i}}=\underset{j}{\sum }\underset{k}{\sum }{\left({\text{Co}}_{i}\right)}_{\text{jk}}\text{, for}\;i=1,\ldots ,M$$

be the sum of all entries in $C_{o_{i}}$ for all symbols in the original observed sequence, and let ${\bar{C}}_{o_{i}}=C_{o_{i}}/c_{o_{i}}$ be the corresponding normalised matrix. Now for each new symbol *o*_*i*_=(*l*_*i*_,*r*_*i*_) in the compressed sequence define

$$c_{o_{i}}=\underset{j}{\sum }\underset{k}{\sum }{\left({\bar{C}}_{l_{i}}{\bar{C}}_{r_{i}}\right)}_{\text{jk}}\text{, for}i=M+1,\ldots ,M^{\prime },$$

and let

$${\bar{C}}_{o_{i}}=\frac{{\bar{C}}_{l_{i}}{\bar{C}}_{r_{i}}}{c_{o_{i}}}\text{, for}\;i=M+1,\ldots ,M^{\prime }.$$

Finally let

$$s_{o_{i}}=\left\{\begin{array}{c} c_{o_{i}},\;\text{, if}\;i=1,\ldots ,M\\ c_{o_{i}}c_{l_{i}}c_{r_{i}},\;\text{, if}\;i=M+1,\ldots ,M^{\prime }. \end{array}\right)$$

Then

(4) $$\begin{array}{l} \alpha_{T}=C_{y_{T^{\;\prime }}^{\;\prime }}C_{y_{T^{\;\prime }-1}^{\;\prime }}\ldots C_{y_{2}^{\;\prime }}C_{1}\\ \;=\left(\overset{T^{\;\prime }}{\underset{t=2}{\prod }}s_{y_{t}}\right){\bar{C}}_{y_{T^{\;\prime }}^{\;\prime }}{\bar{C}}_{y_{T^{\;\prime }-1}^{\;\prime }}\ldots {\bar{C}}_{y_{2}^{\;\prime }}C_{1} \end{array}$$

**Figure 4:**
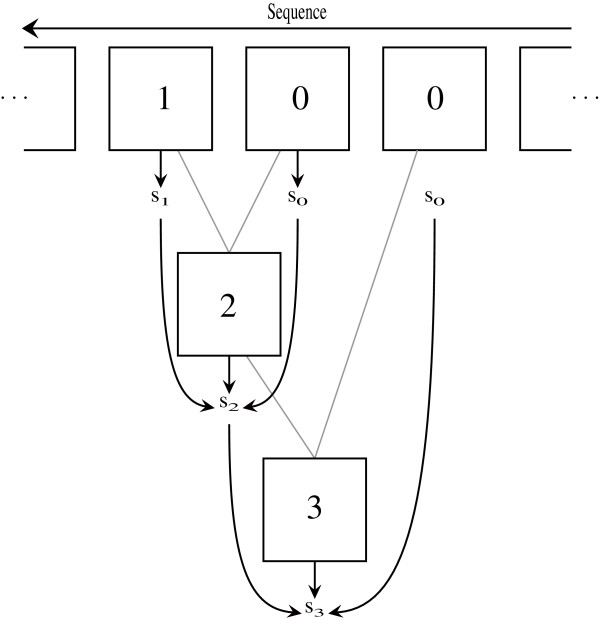
**Dependencies between scaling factors.** Dependencies between scaling factors. The square boxes are the ${\bar{C}}_{o_{i}}$ matrices and *s*_0_,*s*_1_,*s*_2_ and *s*_3_ are scaling factors. The gray lines show the dependencies between the ${\bar{C}}_{o_{i}}$ matrices, while the solid black arrows show how the scaling factors are computed. For example, *s*_0_ is computed directly from ${\bar{C}}_{0}$ as the sum of all its entries. *s*_2_ is the sum of all entries in ${\bar{C}}_{2}$ times *s*_1_ and *s*_0_.

Thus to handle the underflow in the first stage, we compute $s_{o_{i}}$ along with ${\bar{C}}_{o_{i}}$ for *i*=1,…*M*^′^ (see Figure 4) and compute the product above in the second stage of the likelihood computation.

However, the ${\bar{C}}_{o_{i}}$ matrices still only contain values between 0 and 1, and their product will therefore still tend towards 0 exponentially fast, causing underflow. To prevent this we introduce a scaling factor *d*_*i*_ for each of the *T*^′^−1 matrix-vector multiplications in (4), set to be the sum of the entries in the resulting vector. Each *d*_*i*_ is used two times: First we normalise the corresponding resulting vector by dividing each entry by *d*_*i*_, and next we use it to restore the correct result at the end of the computations. Assume that ${\bar{\alpha }}_{T}$ is the result of the *T*^′^−1 normalised matrix-vector multiplications. Then

$$\alpha_{T}=\left(\overset{T^{\prime }}{\underset{t=2}{\prod }}s_{y_{t}}\right)\left(\overset{T^{\prime }-1}{\underset{j}{\prod }}d_{j}\right){\bar{\alpha }}_{T},$$

and we can compute the final likelihood as

$$\begin{array}{lll} \mathrm{Pr}\left(Y_{1:T}\;|\;\lambda \right) & =\underset{i}{\sum }\alpha_{T}(i)\; & \;\\ & =\underset{i}{\sum }\left(\overset{T^{\prime }}{\underset{t=2}{\prod }}s_{y_{t}}\right)\left(\overset{T^{\prime }-1}{\underset{j}{\prod }}d_{j}\right){\bar{\alpha }}_{T}(i)\; & \;\\ & =\left(\overset{T^{\prime }}{\underset{t=2}{\prod }}s_{y_{t}}\right)\left(\overset{T^{\prime }-1}{\underset{j}{\prod }}d_{j}\right)\underset{i}{\sum }{\bar{\alpha }}_{T}(i)\; & \;\\ & =\left(\overset{T^{\prime }}{\underset{t=2}{\prod }}s_{y_{t}}\right)\left(\overset{T^{\prime }-1}{\underset{j}{\prod }}d_{j}\right).\; & \; \end{array}$$

Notice, however, that we now risk getting an underflow when computing these products if *T*^′^ is big. We handle this by working in log-space. Define

$${\tilde{s}}_{o_{i}}=\left\{\begin{array}{ll} \log(c_{o_{i}})\; & ,\text{if}i=1,\ldots ,M\\ \log(c_{o_{i}})+{\tilde{s}}_{l_{i}}+{\tilde{s}}_{r_{i}}\; & ,\text{if}i=M+1,\ldots ,M^{\prime } \end{array}\right)$$

and

$${\tilde{d}}_{i}=\log(d_{i})\;,\text{for}i=1,\ldots ,T^{\prime }-1.$$

Then

$$\log\left(\mathrm{Pr}\left(Y_{1:T}\;|\;\lambda \right)\right)=\left(\overset{T^{\prime }}{\underset{t=2}{\sum }}{\tilde{s}}_{y_{t}}\right)\left(\overset{T^{\prime }-1}{\underset{j}{\sum }}{\tilde{d}}_{j}\right).$$

### Practical implementation details

In our implementation of the preprocessing phase described above, we simply build a map symbol2pair, mapping each new alphabet symbol *o*_*i*_ to its two constituents (*l*_*i*_,*r*_*i*_). In each scan every pair of symbols is counted, and the most frequent pair in the previous round is replaced by a new symbol. The data structure being saved in the end is symbol2pair along with two other maps: nstates2alphabetsize and nstates2seq. The map nstates2alphabetsize maps each *Nmini* to the size of the alphabet *Mj*′ after *j* iterations, where *j* is the number of iterations determined by the stopping criterion. The map nstates2seq maps $N_{\min}^{i}$ to the resulting sequence after *j* rounds of compression. These maps can be saved to disk for later use along with the original observed sequence.

Given a specific HMM with *N* states, the first stage of the actual computation of the likelihood builds a list, symbol2matrix, containing the ${\bar{C}}_{o_{i}}$ matrices, and a list of scaling values, symbol2scale, containing the ${\tilde{s}}_{i}$ values. These are computed in $O\;\left(M^{\prime }N^{3}\right)$ time in an iteration over the first *M*^′^ symbols in the symbol2pair map, where *M*^′^ is the alphabet size saved in nstates2alphabetsize in the preprocessing procedure. In the second stage log(Pr(*Y*_1:*T*_ | *λ*)) is computed in $O\;\left(T^{\prime }N^{2}\right)$ time by

(5) $$\begin{array}{l} \log\left(\mathrm{Pr}\left(Y_{1:T}\;|\;\lambda \right)\right)\\ =\left(\overset{T^{\prime }}{\underset{t=2}{\sum }}{\tilde{s}}_{y_{t}}\right)\left(\overset{T^{\prime }-1}{\underset{j}{\sum }}{\tilde{d}}_{j}\right){\bar{C}}_{y_{T^{\prime }}^{\prime }}{\bar{C}}_{y_{T^{\prime }-1}^{\prime }}\ldots {\bar{C}}_{y_{2}^{\prime }}C_{1}, \end{array}$$

using the two maps created in the first stage. To obtain maximal performance, we use a BLAS implementation for C++ to perform the series of matrix multiplication.

Our implementation uses O(Tk) space in the preprocessing phase and $O\;\left(N^{2}(T^{\prime }+M^{\prime })\right)$ space in the actual computation, where *k* is the number of *N*_*min*_ values supplied by the user, *N* is the number of states in the HMM used in the actual computation, and *M*^′^ is the number of symbols in the extended alphabet corresponding to *N* in nstates2alphabetsize. If the preprocessed data structure is saved to disk, it will take up O(Tk) space.

We have also implemented the algorithm in a parallelised version. In this version, stage 2 is parallelised much like the implementation in parredHMMlib [11], where the series of matrix multiplications in (5) is split into a number of blocks which are then processed in parallel. Stage 1 can clearly also be parallelised by computing independent ${\bar{C}}_{o_{i}}$ matrices in parallel. However, we found that this does not work well in practice, as the workload in stage 1 is not big enough to justify the parallelization. Stage 1 is therefore not parallelised in the library. The parallelisation of stage 2 gives the greatest speedup for long sequences that are not very compressible. This is because the parallelisation in general works best for long sequences [11], and if the input sequence is very compressible then the compressed sequence will be short and more work will be done in the non-parallelised stage 1. The experiments presented in the next section have all been run single-threaded to get a clearer picture of how the runtime of the basic algorithm is influenced by the characteristica of the input sequence and model. But in general a slightly faster running time can be expected if parallelisation is enabled, especially for long sequences of high complexity.

## Results and discussion

We have implemented the above algorithms in a C++ library named zipHMM. The code provides both a C++ and a Python interface to the functionality of reading and writing HMMs to files, preprocessing input sequences and saving the results, and computing the likelihood of a model using the forward algorithm described in the previous section. The library uses BLAS for linear algebra operations and pthreads for multi-threaded parallelisation.

### Using the library

The library can be used directly in C++ programs or through Python wrappers in scripts.

#### Using zipHMM from C++

**Figure 5:**
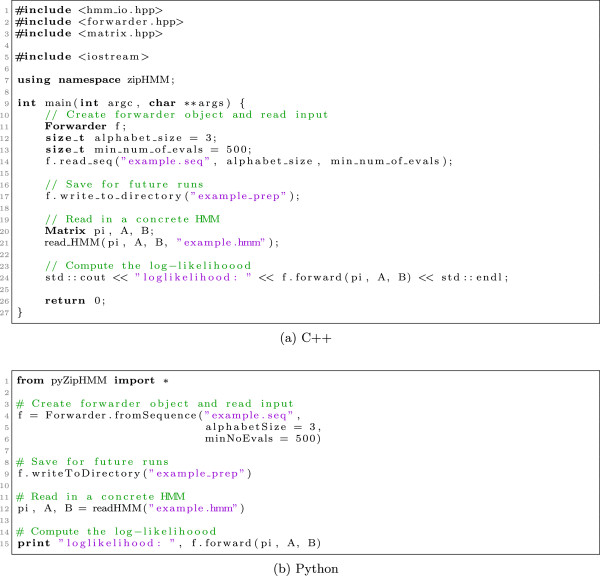
**Using the library in C++(a)and Python(b).**

When using the library in C++ the most important objects are from the Forwarder class, which is responsible for both preprocessing sequences, reading and writing the preprocessed data structure, and for computing the likelihood of a hidden Markov model. The code snippet in Figure 5(a) shows a complete C++ program that reads in an input sequence, f.read_seq(...), preprocess it (as part of reading in the sequence), stores the preprocessed structure to disk, f.write_to_directory(...), reads in an HMM from disk, read_HMM(...), and computes the likelihood of the HMM, f.forward(...).

The sequence reader takes the alphabet size as parameter. This is because we cannot necessarily assume that the observed symbols in the input sequence are all the possible symbols the HMM can emit, so we need to know the alphabet size explicitly. It furthermore takes an optional parameter in which the user can specify an estimate, *e*, of the number of times the preprocessing will be reused. The default value of this parameter is 1.

If the preprocessed sequence is already stored on disk, we can simply read that instead like this:

Forwarder f;number_of_states = 4;f.read_from_directory(“example_preprocessed”, number_of_states);

This will cause the saved sequence matching *N*_*min*_≤4 to be read from the directory example_preprocessed together with additional information on the extended alphabet used in this sequence.

In the library, HMMs are implicitly represented simply by a vector and two matrices, the *π* vector of initial state probabilities and the transition, *A*, and emission, *B*, matrices as described in the Implementation section. These are all represented in a Matrix class, and in the program in Figure 5(a) these are read in from disk. They can also be directly constructed and manipulated in a program. In our own programs we use this, together with a numerical optimisation library, to fit parameters by maximising the likelihood.

The f.forward(...) method computes the likelihood sequentially using the preprocessed structure. To use the multi-threaded parallelisation instead, one simply uses the f.pthread_forward(...) function, with the same parameters, instead.

For completeness the library also offers implementations of the Viterbi and posterior decoding algorithms. To use these in C++ the headers viterbi.hpp and posterior_decoding.hpp should be included and the functions viterbi(...) and posterior_decoding(...) should be called as described in the README file in the library.

#### Using zipHMM from Python

All the C++ classes in the library are wrapped in a Python module so the full functionality of the zipHMM is available for Python scripting using essentially the same API, except with a more Python flavour where appropriate, e.g. reading in data is handled by returning multiple values from function calls instead of pass-by-reference function arguments and with a more typical Python naming convention. Figure 5(b) shows the equivalent of the C++ code in Figure 5(a) in Python.

### Performance

To evaluate the performance of zipHMM we performed a number of experiments using a hidden Markov model previously developed to infer population genetics parameters of a speciation model. All experiments were run on a machine with two Intel Sandy Bridge E5-2670 CPUs, each with 8 cores running at 2.67GHz and having access to a 64Gb main memory. We compare the performance of our forward algorithm to the performance of the implementations of the forward algorithm in HMMlib [19] and in parredHMMlib [11] and to a simple implementation of equation (3) using BLAS to perform the series of matrix-vector multiplications. HMMlib is an implementation that takes advantage of all the features of a modern computer, such as SSE instructions and multiple cores. The individual features of HMMlib can be turned on or off by the user, and we recommend only enabling these features for HMMs with large state spaces. In all our experiments we enabled the SSE parallelisation but used only a single thread. The parredHMM library implements equation (3) as a parallel reduction, splitting the series of matrix multiplications into a number of blocks and processing the blocks in parallel. The parredForward algorithm was calibrated to use the optimal number of threads.

For performance evaluation we wanted to evaluate how well the new algorithm compares to other optimised forward implementations, evaluate the trade-off between preprocessing and computing the likelihood, and explore how the complexity of the input string affects the running time.

**Figure 6:**
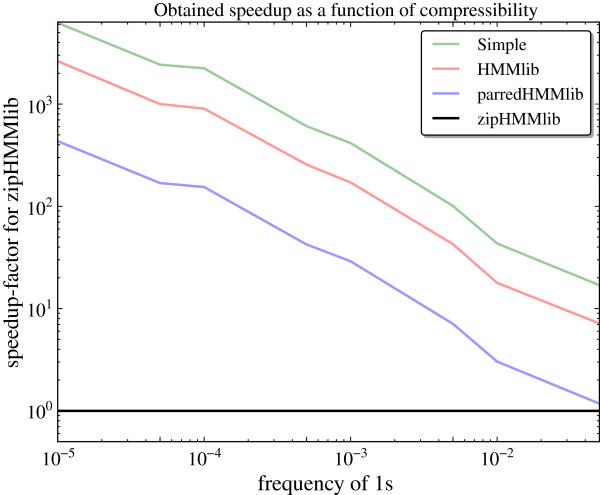
**Speedup vs. sequence complexity.** The speedup factor decreases linearly with the sequence complexity.

Our new implementation of the forward algorithm is expected to perform best on strings of low complexity because they are more compressible. To investigate this we measured the per-iteration running time of the forward algorithm for parredHMMlib, HMMlib and the simple implementation of equation (3) on random binary sequences (over the alphabet {0,1}) of length *L*=10^7^ with the frequency of 1s varying from 0.0001 to 0.05, and divided it by the per-iteration running time for zipHMMlib (excluding the preprocessing time) to obtain the speedup factor. This experiment is summarised in Figure 6, where we note that the speedup factor decreases linearly with the complexity of the input sequence; however, speedup factors of more than two orders of magnitude are obtained for less complex sequences, and even for sequences of low complexity a (modest) speedup is obtained.

In the rest of our experiments, we used a coalescent hidden Markov model (CoalHMM) from [17] together with real genomic data for the experiments. A CoalHMM [7,8,12] exploits changing gene-trees along a genomic alignment to infer population genetics parameters. The “Isolation-with-Migration” CoalHMM from [17] considers a pairwise alignment as its observed sequence and a discretisation of the time to the most recent common ancestor, or “coalescence time”, of the aligned sequences as its hidden states. The coalescence time can change from any point to another, so the transition matrix of the CoalHMM is fully connected, and the number of hidden states can be varied depending on how fine-grained we want to model time. Varying the number of states lets us explore the performance as a function of the number of states. The performance as a function of the length of the input was explored by using alignments of varying length. Finally, to explore how the complexity of the string affects the performance we used alignments of sequences at varying evolutionary distance, since closer related genomes have fewer variable sites and thus the alignments have lower complexity. The CoalHMM model uses a Jukes-Cantor model in its emission probabilities and thus only distinguishes between if a specific site has two identical nucleotides or two different nucleotides in the alignment. We therefore also varied the complexity of the strings by compressing either the actual sequence alignment or summarising it as an alphabet of size three, {0,1,2}, for identical sites, differing sites, or missing data/gaps. This way we obtain sequences with alphabets of size *M*=3 ({0,1,2}), *M*=16 (full alignments over {*A*,*C*,*G*,*T*}×{*A*,*C*,*G*,*T*}, where columns with missing data or gaps were deleted) and *M* = 25 (full alignments over {*A*,*C*,*G*,*T*,*N*}×{*A*,*C*, *G*,*T*,*N*}, where *N* is either missing data or a gap).

For all experiments we trained the model using the Nelder-Mead-simplex algorithm and measured the preprocessing time and total optimisation time, and the expected number of likelihood computations was set to *e*=500.

**Figure 7:**
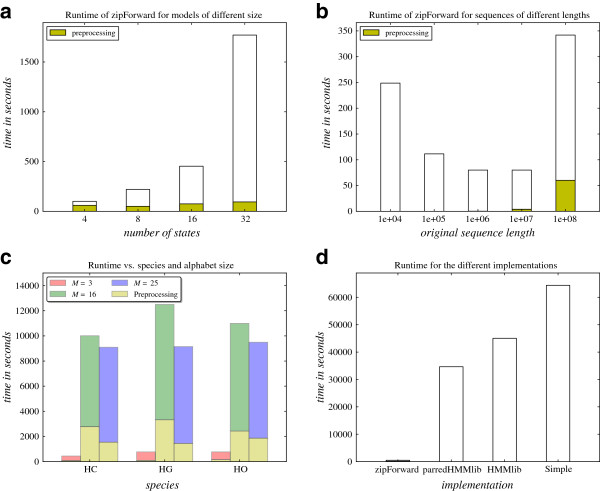
Performance experiments with the CoalHMM framework, showing the running time of zipHMMlib as a function of (a) model size, (b) sequence length, (c) sequence complexity, and (d) compared to other implementations.

Figure 7(a) shows how the performance of zipForward changes, when the size of the model is increased. We note that the total time, as expected, depends very heavily on the number of states (the time complexities of stage 1 and 2 are qubic and quadratic in the number of states, respectively), while the time used on preprocessing, also as expected, varies very little and shows no clear pattern as the number of states is increased.

Figure 7(b) shows how the runtime for training the CoalHMM with zipForward changes when the sequence length is varied. We expected the runtime to increase with the sequence length, however this is not what the results show for the shorter sequences. This is due to the optimisation procedure, which required more iterations of the likelihood computation for the shorter sequences than for the longer sequences. For the longest sequences the runtime grows sublinearly, which was expected, since longer sequences often compress relatively more than shorter sequences.

We expected alignments of sequences at short evolutionary distance to be more compressible than alignments of sequences at longer evolutionary distance, and therefore expected the training procedure to be faster for alignments of sequences at short evolutionary distance. We recognise this in Figure 7(c) except for the sequences with *M*=16, where the human-orangutan alignment was processed faster than the human-gorilla alignment. However, this was caused by the trade-off between the time for the preprocessing and the time for the actual training procedure: the preprocessing procedure took significantly longer time for the human-gorilla alignment (because it was more compressible) than for the human-orangutan alignment, but this extra time was not all gained back in the training procedure, although the compressed sequence indeed was shorter (the per-iteration running time for the human-gorilla alignment was 7.913*s* and 8.488*s* for the human-orangutan alignment).

We also expected the total time of the training procedure to increase as the number of symbols in the initial alphabet was increased, because sequences with small initial alphabets are expected to be more compressible than sequences with larger initial alphabets. But as Figure 7(c) shows, the sequences with an initial alphabet of size *M*=25 were processed faster than the sequences with an initial alphabet of size *M* = 16. This is again caused by the optimisation procedure, which converges faster for the sequences with *M*=25 than for the sequences with *M*= 16 (e.g. for the human-gorilla alignments, the number of evaluations of the likelihood were 860 and 1160 for *M*=25 and *M*=16, respectively). This may be a result of the sequences with *M*=25 containing more information than the sequences with *M*=16. The lengths of the compressed sequences and the per-iteration running times match our expectations, and for the sequences with *M*=3 and *M*=25, which contain the same data, the algorithm behaves as expected.

Figure 7(d) shows the performance of the four different implementations of the forward algorithm. Each of the four algorithms was used to train the CoalHMM on an alignment of the entire human and chimpanzee chromosome 1, using an HMM with 16 states and an initial alphabet of size 3. The training procedure was finished in 7.4 minutes (446 seconds) using zipForward and including the preprocessing time. This gives a speedup factor of 77.7 compared to the previously fastest implementation using parredHMMlib, which used 9.6 hours (34,657 seconds). It is therefore evident that zipForward is clearly superior to the three other implementations on this kind of input. The time used per iteration of the likelihood computation was 0.5042 seconds for zipHMMlib, while it was 46.772 seconds for parredHMMlib, leading to a speedup of a factor 92.8 on the actual optimization procedure (excluding preprocessing time). Repeating the same experiment on full alignments over alphabets of size 16 and 25 (not shown here), where zipForward clearly performs worse than for sequences with alphabets of size 3 (see Figure 7(c)), we still obtained total speedup factors of 4.4 for both experiments.

## Conclusions

We have engineered a variant of the HMM forward algorithm to exploit repetitions in strings to reduce the total amount of computation, by exploring shared sub-expressions. We have implemented this in an easy to use C++ library, with a Python interface for use in scripting, and we have demonstrated that our library can be used to achieve speedups of 4 - 78 factors for realistic whole-genome analysis with a reasonably complex hidden Markov model.

## Availability and requirements

**Project name:** zipHMM**Project home page:**http://birc.au.dk/software/ziphmm/**Operating system(s):** Platform independent**Programming language:** C++, Python**License:** LGPL

## Competing interests

The authors declare that they have no competing interests.

## Authors’ contributions

AS, MK and TM developed the algorithms. AS implemented the library. AS, CNSP and TM designed experiments. AS, CNSP and TM drafted the manuscript. All authors read and approved the final manuscript.
